# Effects of High-Definition Transcranial Direct Current Stimulation Over the Primary Motor Cortex on Cold Pain Sensitivity Among Healthy Adults

**DOI:** 10.3389/fnmol.2022.853509

**Published:** 2022-03-18

**Authors:** Xiaoyun Li, Xinxin Lin, Junjie Yao, Shengxiong Chen, Yu Hu, Jiang Liu, Richu Jin

**Affiliations:** ^1^School of Psychology, Shenzhen University, Shenzhen, China; ^2^Medical Rehabilitation Center, Shenzhen Prevention and Treatment Center for Occupational Diseases, Shenzhen, China; ^3^Department of Computer Science and Engineering, Southern University of Science and Technology, Shenzhen, China

**Keywords:** high-definition transcranial direct current stimulation (HD-tDCS), primary motor cortex (M1), pain, analgesia, cold pain sensitivity

## Abstract

Some clinical studies have shown promising effects of transcranial direct current stimulation (tDCS) over the primary motor cortex (M1) on pain relief. Nevertheless, a few studies reported no significant analgesic effects of tDCS, likely due to the complexity of clinical pain conditions. Human experimental pain models that utilize indices of pain in response to well-controlled noxious stimuli can avoid many confounds that are present in the clinical data. This study aimed to investigate the effects of high-definition tDCS (HD-tDCS) stimulation over M1 on sensitivity to experimental pain and assess whether these effects could be influenced by the pain-related cognitions and emotions. A randomized, double-blinded, crossover, and sham-controlled design was adopted. A total of 28 healthy participants received anodal, cathodal, or sham HD-tDCS over M1 (1 mA for 20 min) in different sessions, in which montage has the advantage of producing more focal stimulation. Using a cold pressor test, several indices reflecting the sensitivity to cold pain were measured immediately after HD-tDCS stimulation, such as cold pain threshold and tolerance and cold pain intensity and unpleasantness ratings. Results showed that only anodal HD-tDCS significantly increased cold pain threshold when compared with sham stimulation. Neither anodal nor cathodal HD-tDCS showed significant analgesic effects on cold pain tolerance, pain intensity, and unpleasantness ratings. Correlation analysis revealed that individuals that a had lower level of attentional bias to negative information benefited more from attenuating pain intensity rating induced by anodal HD-tDCS. Therefore, single-session anodal HD-tDCS modulates the sensory-discriminative aspect of pain perception as indexed by the increased pain threshold. In addition, the modulating effects of HD-tDCS on attenuating pain intensity to suprathreshold pain could be influenced by the participant’s negative attentional bias, which deserves to be taken into consideration in the clinical applications.

## Introduction

Non-invasive brain stimulation techniques, such as transcranial magnetic stimulation and transcranial electrical stimulation, are neuromodulation approaches that can regulate the cortical activity (Nitsche et al., 2008; Lefaucheur et al., 2017). Transcranial direct current stimulation (tDCS) is the most commonly used transcranial electrical stimulation technique, due to its relatively small-size, low-cost, ease-of-use, and safety characteristics (Lefaucheur et al., 2017). The primary mechanism of tDCS is considered to induce polarity-dependent shifts in the resting membrane potentials, thereby, modulating cortical excitability and neuronal spontaneous firing rate (Creutzfeldt et al., 1962; Purpura and McMurtry, 1965). In general, anodal stimulation results in neuronal depolarization and increases cortical excitability, whereas cathodal stimulation causes neuronal hyperpolarization and decreases cortical excitability (Nitsche and Paulus, 2000; Nitsche et al., 2008).

Previous studies have shown tDCS effects on attenuating pain perception in experimental pain and clinical pain conditions, such as neuropathic pain, fibromyalgia, and migraine (Fregni et al., 2007; Lefaucheur et al., 2008; Nitsche et al., 2008; Luedtke et al., 2012b; Mylius et al., 2012). Applying 20-min anodal tDCS over the primary motor cortex (M1) is recommended for pain relief in the evidence-based guidelines (Lefaucheur et al., 2017). Meta-analysis showed that tDCS over M1 has small to moderate analgesic effects on pain threshold in both healthy and chronic pain populations (Vaseghi et al., 2015; Giannoni-Luza et al., 2020). However, some studies reported no significant analgesic effects of tDCS as compared with sham condition (Jürgens et al., 2012; Luedtke et al., 2012a,^2015^). For instance, a single-blinded crossover study found that tDCS over M1 was failed to modulate pain threshold and ratings to suprathreshold heat stimuli among healthy volunteers (Jürgens et al., 2012). In addition, anodal tDCS over M1 did not significantly relieve pain and disability for 135 patients with chronic low back pain (Luedtke et al., 2015). These heterogeneous results lead to the question of whether the active tDCS stimulation of M1 is effective for pain modulation.

Most studies that investigated the effects of tDCS over M1 upon pain perception have adopted the conventional montage with the target electrode placed at the M1 and the reference electrode placed at the contralateral supraorbital area. Nevertheless, the spatial distribution of the electrical field for conventional tDCS configurations has been critically discussed. Conventional tDCS stimulation modulates cortical activation in a large cortical area beyond the cortical region underlying the target electrode (Lang et al., 2005; Nitsche et al., 2007). In addition, modeling studies provide evidence that electric fields produced by conventional tDCS montage are highly diffuse, and the target area does not directly receive the largest current density (Datta et al., 2009; Bikson et al., 2010). Relative to conventional tDCS, 4 × 1 high-definition tDCS (HD-tDCS) montage with smaller electrodes allows to restrict the current flow between the central and return electrodes, thereby, providing a more focal stimulation in the target area (Kuo et al., 2013; Villamar et al., 2013). Thus, HD-tDCS seems to overcome one of the main limitations of conventional tDCS by improving the spatial precision of stimulation.

In light of the advantages of HD-tDCS, some studies attempted to assess whether HD-tDCS targeted on M1 can effectively alleviate clinical pain (Villamar et al., 2013; Castillo-Saavedra et al., 2016). For instance, a phase II open-label trial reported that 15 sessions (median number) of HD-tDCS over M1 could achieve a 50% pain reduction in fibromyalgia patients (Castillo-Saavedra et al., 2016). Indeed, there are inevitably some confounding factors in the clinical pain population, such as pain comorbidity of anxiety and depression. Human experimental pain models allow to provide noxious stimuli with standardized intensity and to rigorously measure pain responses with a high level of precision. Noxious stimuli (e.g., cold pressor) of the intensity and modality can be applied in a controlled laboratory setting while other variables of interest are systematically manipulated. In addition, indices of pain perception in response to the well-controlled noxious stimuli can be measured with psychophysical methods. Thus, human experimental pain models are often used to measure pain sensitivity and avoid many confounds presented in the clinical data. A recent study showed that HD-tDCS over M1 was delivered across 3 days among the healthy individuals, but was failed to modulate somatosensory and pain sensitivity (Kold and Graven-Nielsen, 2021). In this study, somatosensory detection and pain thresholds were measured, which mainly reflect the sensory-discriminative aspect of pain perception (Rainville et al., 1992). It remains unclear whether HD-tDCS can modulate the affective-motivation aspect of pain, such as pain tolerance.

Pain perception is greatly dependent upon psychological factors, such as pain-related cognitions and emotions (Bushnell et al., 2013). These psychological factors (e.g., pain catastrophizing and fear of pain) can also predict the outcomes in clinical interventions of clinical pain (Werneke et al., 2009; Mankovsky et al., 2012; Sparkes et al., 2015; Burns et al., 2017; Sharifzadeh et al., 2017). For example, greater pain catastrophizing predicts a worse response to opioid analgesics for patients with chronic low back pain (Burns et al., 2017) and less pain reduction after spinal cord stimulation treatment for patients with chronic neuropathic pain (Sparkes et al., 2015). Moreover, more fear of pain is associated with worse outcomes in physical rehabilitation therapy for patients with low back pain (Werneke et al., 2009). Since the effectiveness of pain intervention is greatly influenced by pain-related cognitions and emotions, it is likely that these psychological factors could influence the analgesic effects of tDCS. Understanding the underlying moderating factors of analgesia induced by tDCS may help to develop tDCS protocols for precision medicine.

Cold pressor pain, induced by submerging a non-dominant hand into cold water, is a well-validated test to mimic clinical pain, because of the more sustained and higher level of pain intensity and unpleasantness that it evokes (Rainville et al., 1992). It shows excellent experimental reliability and validity in assessing cold pain sensitivity (Ehrlich et al., 2003). Here, the present study used the cold pressor test and investigated the effects of single-session HD-tDCS over M1 on cold pain sensitivity among healthy participants. Adopting a randomized, double-blinded, crossover, and sham-controlled design, pain sensitivity was measured immediately after anodal, cathodal, and sham HD-tDCS targeted on the M1. We hypothesized that when compared with sham stimulation, active HD-tDCS over M1 could increase cold pain threshold and tolerance but decrease pain intensity and unpleasantness ratings. In addition, we hypothesized that the effectiveness of active HD-tDCS on cold pain sensitivity could be influenced by pain-related cognitions and emotions.

## Materials and Methods

### Participants

*A priori* power analysis using G*Power software was conducted to determine the appropriate sample size for a within-participant design with two factors (2 × 3 = 6 conditions). It yielded a sample size of *n* = 28 to detect a medium effect size of *f* = 0.25 at a standard error probability of α = 0.05 with a power of 0.95. Therefore, we recruited 28 participants [14 women; age: mean ± standard error of the mean (SEM) = 23.07 ± 0.34 years) to participate in this study. All participants were right-handed, had a normal or corrected-to-normal vision, and were free from any contraindications for tDCS application. No participant reported any medical condition associated with acute or chronic pain, cardiovascular or neurological diseases, psychiatric disorders, or current use of any medication, or in menstrual period. All participants gave their written informed consent before the experiments according to the Declaration of Helsinki. All experimental procedures were approved by the local research ethics committee.

### Questionnaires

Before the experiment, all participants were instructed to complete the pain-related questionnaires that measured their cognitions and emotions to pain. Specifically, the Pain Sensitivity Questionnaire (PSQ) was used to assess subjective pain perception of painful situations in daily life (Ruscheweyh et al., 2009). The Fear of Pain Questionnaire (FPQ; McNeil and Rainwater, 1998) and the Pain Catastrophizing Scale (PCS; Sullivan et al., 1995) were administered to assess their thoughts, attitudes, and beliefs toward pain. The Pain Vigilance and Awareness Questionnaire (PVAQ) was used to measure awareness, consciousness, vigilance, and observation of pain (McCracken, 1997). The Attention to Positive and Negative Information Scale (APNI; Noguchi et al., 2006) was adopted to examine the individual attentional bias to positive or negative information, which consisted of two subscales (Attention to Positive Information, API; Attention to Negative Information, ANI).

### General Experimental Procedure

**FIGURE 1:**
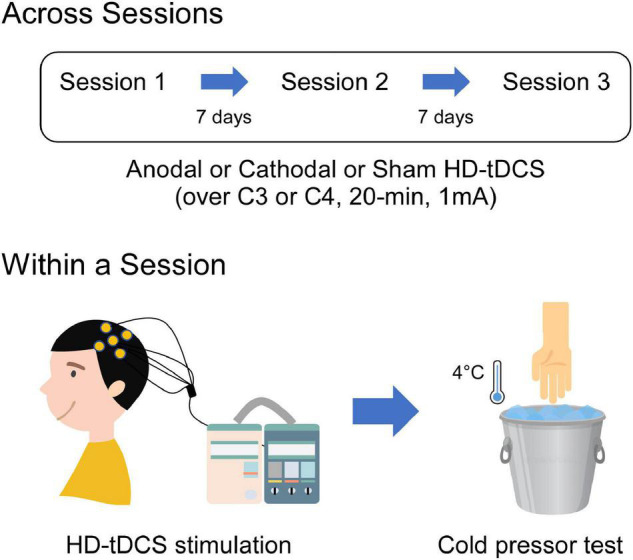
Schematic illustration of the experimental procedures. Each participant visited three sessions which comprised single-session tDCS intervention (1 mA, 20 min) followed by a cold pressor test. Three sessions were separated by 7 days to avoid the possible carryover effects due to the tDCS stimulation. During the tDCS intervention, participants received HD-tDCS targeted on the M1 using anodal, cathodal, or sham stimulation. HD-tDCS electrodes were placed according to the International 10–20 System, with the central electrode placed at C3 or C4 and the return electrodes placed at FC5, FC1, CP1, CP5 or FC6, FC2, CP2, CP6, respectively. During the cold pressor test, sensitivity to cold pain was measured by instructing participants to immerse their both hands (contralateral and ipsilateral to the HD-tDCS target side) into the cold-water apparatus of 4°C.

This study was a randomized, double-blinded, crossover, and sham-controlled design. Two experimenters were involved in this study, with one as the tDCS administrator and the other as the pain-test assessor. The tDCS administrator was responsible for the generation of the random allocation sequence and the delivery of the tDCS intervention, who was not involved in any data collection and analysis. As shown in Figure 1, each participant attended three sessions and underwent a single session of anodal, cathodal, and sham HD-tDCS targeted on the left or right M1, which were followed by a cold pressor test. Sessions were separated by at least 1 week to prevent any carryover effects. The order and the stimulated site of tDCS intervention were counterbalanced and randomly assigned to the participants. Therefore, each participant received three sessions of HD-tDCS (anodal, cathodal, and sham) with the target region on either left or right M1, which was kept constant across the three sessions.

#### High-Definition Transcranial Direct Current Stimulation

**FIGURE 2:**
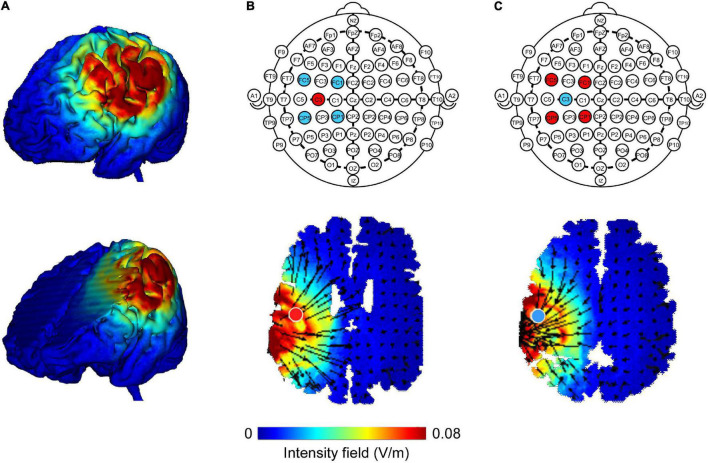
Finite element models of high-definition transcranial direct current (HD-tDCS) of the left M1. Cortical surface and deep brain structure plots illustrate electric field magnitude (**A**; directionless; blue color represents zero electric field and the red color represents peak magnitude). HD-tDCS electrodes were placed according to the International 10–20 System. The central electrode was placed at C3 with surrounding return electrodes that are located at FC1, FC5, CP5, and CP1 (red color represents anode, blue color represents cathode). Anodal **(B)** and cathodal **(C)** modeling showed a similar current field intensity but with current flow in opposite directions. The direction of the current flow is indicated by black arrows. A field intensity range of 0.00–0.08 V/m was used.

A 4 × 1 Multichannel Stimulation Adaptor (Model 4 × 1-C3A; Soterix Medical Inc., New York, NY, United States) was employed to deliver 1 mA direct current to the scalp *via* Ag-AgCl sintered ring electrodes (EL-TP-RNG Sintered; Stens Biofeedback Inc., San Rafael, CA, United States) (Minhas et al., 2010). The 4 × 1 ring montage consisted of one central electrode placed at the M1 (C3 or C4) based on the International 10–20 System, and the four return electrodes were surrounded the central electrode at a center-to-center distance of 3.5 cm. When stimulating the left M1, the central electrode was placed on C3, while the four return electrodes were placed on FC1, FC5, CP5, and CP1. When stimulating the right M1, the central electrode was placed on C4, and the four return electrodes were placed on FC2, FC6, CP6, and CP2. Previous studies have confirmed that the position of the electrode at C3 or C4 corresponds approximately to the location of the left or right M1 (Edwards et al., 2013). HD-Explore software (Version 2.3, Soterix Medical, New York, NY, United States) was used to confirm the focality of electric fields induced by HD-tDCS (Figure 2). The identical montage setting was used for the anodal, cathodal, and sham stimulation. Impedance values were measured for each of the five electrodes and were all verified to be <1 quality unit. For anodal and cathodal stimulations, the current ramped up from 0 to 1 mA in 30 s and was then constantly given for 20 min, with a 30 s ramp-down time period at the end of the stimulation. For sham stimulation, the current ramped up to 1 mA over 30 s, prior to being ramped down over the next 30 s to 0 mA, where its stimulation protocol was still maintained for 20 min. At the end of the stimulation, the current was again ramped up to 1 mA over 30 s. Participants were blinded to the type of HD-tDCS stimulation and the device was kept out of their sight during the experiment. At the end of each session, participants completed a questionnaire regarding blinding efficiency and potential adverse effects caused by the HD-tDCS stimulation, such as itching, pain, or skin irritation (Antal et al., 2010; Brunoni et al., 2011).

#### Cold Pressor Test

Immediately after HD-tDCS intervention, a cold pressor test was conducted to assess individual cold pain sensitivity. The test was applied to both hands (contralateral and ipsilateral to the HD-tDCS stimulated side), separated by 10 min. The testing order of two hands was counterbalanced and randomly assigned for the participants. Participants were instructed to firstly immerse the hand up to the wrist into a tank with room temperature water at approximately 22°C for 30 s. This was done to ensure that the hand temperature before a cold pressor test was similar across participants. Then, participants were asked to immediately immerse open-hand into a circulating cold-water tank (Type: DX-208, Beijing Changliu Scientific Instrument Co., Ltd.) of 4°C (±0.10). Simultaneously, a stopwatch was activated. Cold pain threshold was defined as the total duration from the onset of hand immersion until the first report of pain perception (in seconds). Cold pain tolerance was defined as the total duration from the onset of hand immersion until the removal of the hand from the cold pressor apparatus (in seconds). Perceived pain intensity and unpleasantness were rated at tolerance, using an 11-point scale ranging from 0 (no pain/unpleasantness) to 10 (unbearable pain/unpleasantness). For the safety concerns, we would instruct the participants to withdraw their hand from the apparatus if the immersion duration reached 3 min. This was not informed to the participants before the cold pressor test.

### Statistical Analysis

All statistical analyses were carried out using the IBM SPSS statistical analysis package (version 22; IBM Corp., Armonk, NY, United States). The blinding of tDCS type was examined using a Cochran’s *Q*-test, which compared the frequency of yes responses across three sessions. Ratings for adverse effects were investigated using the one-way repeated measures analysis of variance (ANOVA) with a factor of tDCS Type (anodal, cathodal, and sham HD-tDCS). To assess possible effects of HD-tDCS on cold pain sensitivity, measures in the cold pressor test (such as cold pain threshold and tolerance, as well as ratings of perceived pain intensity and unpleasantness) were compared using a two-way repeated measures ANOVA with two within-participant factors of tDCS Type (anodal, cathodal, and sham HD-tDCS) and Stimulation Side (hands ipsilateral and contralateral to the HD-tDCS side). When there was a significant main effect or interaction, we performed *post hoc* comparisons. Bonferroni correction was used for multiple-comparison correction. In addition, the relationship between the analgesic effects of HD-tDCS (active HD-tDCS *minus* sham HD-tDCS) and scores on pain-related questionnaires (i.e., PSQ, FPQ, PCS, PVAQ, and APNI) was assessed using Pearson correlation across all participants. This was done to test whether analgesic effects of HD-tDCS were influenced by pain-related cognitions or emotions.

## Results

A total of 28 participants were originally recruited. Three participants failed to complete the three sessions due to either personal issues (*n* = 1) or the equipment failure (*n* = 2). To this end, data from 25 participants were included in the data analysis. The demographic information (including age and gender) and psychometric characteristics (including the PSQ, FPQ, PCS, PVAQ, and APNI) are summarized in Table 1.

**TABLE 1:** Demographic and psychometric characteristics of participants (*n* = 25).

Characteristics	Mean ± SEM
Age (years)	22.92 ± 0.36
Sex (female/male)	13/12
Pain Sensitivity Questionnaire	72.80 ± 4.39
Fear of Pain Questionnaire	101.12 ± 3.24
Pain Catastrophizing Scale	18.2 ± 1.88
Pain Vigilance and Awareness Questionnaire	38.24 ± 1.86
Attention to Positive Information (API)	76.52 ± 0.76
Attention to Negative Information (ANI)	34.72 ± 1.56

Immediately after tDCS intervention, the blinding of stimulation type was evaluated using the questionnaires. The effectiveness of blinding HD-tDCS (i.e., whether the participant believed that they had received active tDCS or not) was analyzed using a Cochran’s *Q*-test. The reports did not differ among the three sessions [χ^2^ (2) = 2.00, *p* = 0.778]. It suggests successful blinding of HD-tDCS. Ratings of adverse events were compared among the three HD-tDCS sessions. As shown in Table 2, ratings of adverse events after HD-tDCS are comparable among the three sessions (*p* > 0.05 for all comparisons), except for ratings of burning sensation (*F*_2,46_ = 5.41, *p* = 0.010, $\eta_{p}^{2}=0.191$). *Post hoc* comparisons showed that participants reported greater burning sensations after cathodal stimulation intervention than after anodal stimulation intervention (*p* = 0.029). Nevertheless, ratings to sham stimulation were not different from anodal or cathodal stimulation (*p* = 0.575 and *p* = 0.110, respectively). It suggests that cathodal HD-tDCS causes more adverse effects on eliciting burning sensation.

**TABLE 2:** Reported adverse effects after anodal, cathodal, and sham HD-tDCS stimulation.

	Anodal	Cathodal	Sham	ANOVA
Headache	0.32 ± 0.16	0.72 ± 0.32	0.63 ± 0.37	*F*_2,46_ = 0.64, *p* = 0.504, $\eta_{p}^{2}=0.027$
Neck pain	0.08 ± 0.08	0.36 ± 0.18	0.13 ± 0.09	*F*_2,46_ = 1.33, *p* = 0.274, $\eta_{p}^{2}=0.055$
Scalp pain	1.56 ± 0.39	1.44 ± 0.41	1.50 ± 0.33	*F*_2,46_ = 0.32, *p* = 0.718, $\eta_{p}^{2}=0.014$
Tingling	2.92 ± 0.46	3.24 ± 0.49	2.96 ± 0.59	*F*_2,46_ = 0.35, *p* = 0.658, $\eta_{p}^{2}=0.015$
Itching	3.24 ± 0.61	3.80 ± 0.59	2.63 ± 0.59	*F*_2,46_ = 2.71, *p* = 0.082, $\eta_{p}^{2}=0.105$
Burning sensation	0.44 ± 0.19	1.88 ± 0.50	0.88 ± 0.44	*F*_2,46_ = 5.41, *p* = 0.010, $\eta_{p}^{2}=0.191$
Skin redness	0.16 ± 0.11	0.68 ± 0.33	0.50 ± 0.30	*F*_2,46_ = 0.66, *p* = 0.462, $\eta_{p}^{2}=0.028$
Sleepiness	2.84 ± 0.40	2.16 ± 0.37	2.92 ± 0.42	*F*_2,46_ = 2.49, *p* = 0.107, $\eta_{p}^{2}=0.098$
Trouble concentrating	2.24 ± 0.38	2.24 ± 0.49	2.13 ± 0.51	*F*_2,46_ = 0.01, *p* = 0.978, $\eta_{p}^{2}=0.001$
Acute mood changes	0.44 ± 0.22	0.68 ± 0.34	0.67 ± 0.36	*F*_2,46_ = 0.22, *p* = 0.733, $\eta_{p}^{2}=0.009$

*Data are expressed as mean ± SEM. Statistics were obtained by applying one-way repeated measures ANOVA with one factor of “Type” (anodal, cathodal, and sham HD-tDCS).*

**FIGURE 3:**
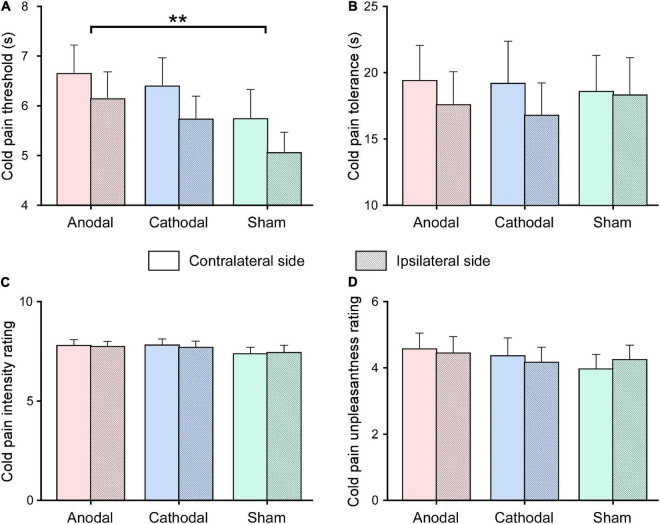
Effects of high-definition transcranial direct current (HD-tDCS) on cold pain sensitivity. Cold pain threshold (measured in seconds; **A**) and tolerance (measured in seconds; **B**), as well as cold pain intensity (measured on the 0–10 scale; **C**) and unpleasantness (measured on the 0–10 scale; **D**) ratings were measured after the application of anodal, cathodal, sham HD-tDCS, for hands contralateral and ipsilateral to the HD-tDCS side. Data are expressed as mean ± SEM. Compared with sham stimulation, the cold pain threshold was significantly increased after anodal HD-tDCS, regardless of whether the cold pain stimulus was delivered to the hand contralateral or ipsilateral to the HD-tDCS side. **p < 0.01, paired-sample *t*-test.

The duration to cold pain threshold and tolerance and ratings to pain intensity and unpleasantness are displayed in Figure 3. Statistics for the effects of HD-tDCS on cold pain sensitivity are summarized in Table 3. Analysis of HD-tDCS effects on cold pain threshold showed a significant main effect of tDCS Type (*F*_2,48_ = 3.83, *p* = 0.035, $\eta_{p}^{2}=0.138$). *Post hoc* paired-sample *t*-tests showed that cold pain threshold was greater after anodal HD-tDCS than after sham stimulation (*p* = 0.008; Figure 3A) but was comparable between cathodal and sham stimulation (*p* = 0.272). The main effect of the Stimulation Side was also significant (*F*_1,24_ = 7.65, *p* = 0.011, $\eta_{p}^{2}=0.242$) such that the cold pain threshold at hand contralateral to the HD-tDCS side was greater than at the ipsilateral side. The interaction was not significant for cold pain threshold (*F*_2,48_ = 0.08, *p* = 0.923, $\eta_{p}^{2}=0.003$). These results suggested that anodal HD-tDCS significantly increased cold pain threshold, in which effect was comparable between hands contralateral or ipsilateral to tDCS target side.

**TABLE 3:** Statistics for cold pain sensitivity.

	Stimulation Side	tDCS Type	Stimulation Side × tDCS Type
Cold pain threshold	*F*_1, 24_ = 7.65*, $\eta_{p}^{2}=0.242$	*F*_2,48_ = 3.83*, $\eta_{p}^{2}=0.138$	*F*_2,48_ = 0.08, $\eta_{p}^{2}=0.003$
Cold pain tolerance	*F*_1, 24_ = 1.52, $\eta_{p}^{2}=0.059$	*F*_2,48_ = 0.19, $\eta_{p}^{2}=0.008$	*F*_2,48_ = 2.13, $\eta_{p}^{2}=0.082$
Pain intensity	*F*_1, 24_ = 0.13, $\eta_{p}^{2}=0.005$	*F*_2,48_ = 1.37, $\eta_{p}^{2}=0.054$	*F*_2,48_ = 0.38, $\eta_{p}^{2}=0.016$
Unpleasantness	*F*_1, 24_ = 0.01, $\eta_{p}^{2}<0.001$	*F*_2,48_ = 0.42, $\eta_{p}^{2}=0.017$	*F*_2,48_ = 1.59, $\eta_{p}^{2}=0.062$

Statistics were obtained by applying a two-way repeated measures ANOVA, with two within-participant factors of Stimulation Side (the hands contralateral and ipsilateral to the HD-tDCS side) and tDCS Type (anodal, cathodal, and sham HD-tDCS). *p < 0.05.

In contrast, repeated measure ANOVA did not show any significant main effects or interaction on pain tolerance, pain intensity, and unpleasantness ratings evoked by cold pressor stimulus (*p* > 0.05 for all comparisons).

**FIGURE 4:**
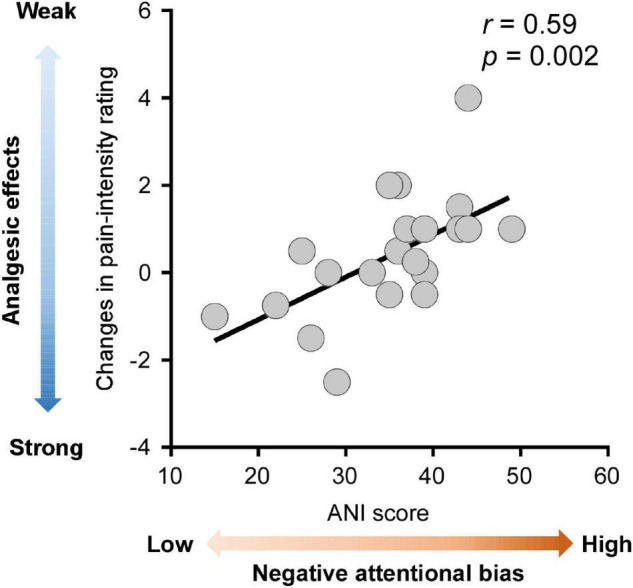
Associations between analgesic effects of anodal high-definition transcranial direct current (HD-tDCS) and negative attentional bias. Analgesic effects of anodal HD-tDCS over M1 on cold pain intensity rating were calculated as the contrast between anodal and sham stimulation condition (anodal HD-tDCS *minus* sham HD-tDCS), with more negative values indicating stronger analgesic effects (colored in darker blue). The negative attentional bias was quantified by the scores on the Attention to Negative Information Scale (ANI), with greater scores on the ANI indicating a higher level of negative attentional bias (colored in darker red). Across all participants, stronger analgesic effects of anodal HD-tDCS were significantly correlated with a lower level of negative attentional bias. Each gray dot in the scatter plots represents a single participant. The black line represents the best linear fit for the data.

Correlation analysis was conducted to determine whether the analgesic effects of HD-tDCS were influenced by pain-related cognitions and emotions. Firstly, cold pain sensitivity measured at hands contralateral and ipsilateral to the HD-tDCS side was grand averaged for each tDCS type (anodal, cathodal, and sham HD-tDCS), thus yielding three values for each pain sensitivity measure and for each participant. Next, the analgesic effects of HD-tDCS were evaluated by calculating the contrast between active and sham stimulation (i.e., anodal *minus* sham or cathodal *minus* sham). A negative value for the pain rating or a positive value for pain threshold and tolerance indicates analgesia induced by active HD-tDCS. Finally, the relationships between the analgesic effects of HD-tDCS and pain-related cognitions/emotions (scores on the PSQ, FPQ, PCS, PVAQ, and APNI) were estimated using Pearson correlation analysis. As shown in Figure 4, the analgesic effect of anodal HD-tDCS on cold pain intensity rating was significantly associated with scores on the ANI (*r* = 0.59, *p* = 0.002). This result suggested that individuals with a lower level of attentional bias to negative information would benefit more from attenuating pain intensity rating induced by anodal HD-tDCS stimulation.

## Discussion

The current study evaluated the effects of single-session HD-tDCS over M1 on cold pain sensitivity among the healthy population. Anodal HD-tDCS showed to be effective in increasing cold pain threshold when compared with sham stimulation. Neither anodal nor cathodal HD-tDCS significantly modulated cold pain tolerance and pain intensity and unpleasantness ratings. Analgesic effects of anodal HD-tDCS on cold pain intensity rating could be influenced by the level of attentional bias to negative information. These results suggested that anodal HD-tDCS over M1 can modulate the sensory-discriminative aspect of experimental pain perception and that analgesic effects of anodal HD-tDCS on the perception of suprathreshold pain may be influenced by psychological factors, such as negative attentional bias.

Single-session 20-min anodal HD-tDCS targeted on the M1, relative to sham stimulation, increased cold pain threshold, regardless of whether a painful stimulus was delivered to the hand contralateral or ipsilateral to the tDCS target site. This finding is in line with Zandieh et al. (2013), which reported that the application of conventional anodal tDCS, but not cathodal tDCS, led to an increment in cold pain threshold. Previous meta-analyses also support our findings and show that anodal tDCS stimulation over M1 increases pain threshold in both healthy (Vaseghi et al., 2014) and clinical pain (Giannoni-Luza et al., 2020) population. However, our results are in contrast with previous studies that reported negative effects of tDCS on cold pain threshold among healthy volunteers (Bachmann et al., 2010; Grundmann et al., 2011; Borckardt et al., 2012; Jürgens et al., 2012; Brasil-Neto et al., 2020; Kold and Graven-Nielsen, 2021). Unlike the conventional pad-based tDCS frequently used in previous studies (Bachmann et al., 2010; Grundmann et al., 2011; Jürgens et al., 2012), we used HD-tDCS montage with multiple smaller electrodes that can provide more focal stimulation on the M1, thereby, increasing the credibility of activating M1 (Nitsche et al., 2007; Kuo et al., 2013). A few studies did employ the HD-tDCS stimulation over M1 but also reported little or marginal effect on cold pain threshold (Borckardt et al., 2012; Brasil-Neto et al., 2020; Kold and Graven-Nielsen, 2021). Most of these studies measured cold pain sensitivity before and after the tDCS intervention, which likely induce habituation or sensitization to the noxious stimulus. As the participants were repeatedly exposed to the same assessments with a relatively short interval (i.e., 15–20 min), the novelty and salience of noxious stimulus would be reduced along with the test progress, which may confound with the analgesic effects of tDCS (Kold and Graven-Nielsen, 2021).

Although previous studies have reported analgesic effects of M1-tDCS in both experimental and clinical pain settings (Lefaucheur et al., 2008; Giannoni-Luza et al., 2020), the underlying mechanisms still remain unclear. One of the hypotheses proposes that M1-tDCS induced analgesia through the inhibition of the nociceptive ascending pathway at the spinal cord level by activating the endogenous pain inhibitory pathway (García-Larrea et al., 1999; Giannoni-Luza et al., 2020). Neuroimaging studies provide evidence that motor cortex stimulation triggers activation in the ventral-lateral thalamus, leading to a cascade of events in medial thalamus, anterior cingulate/orbitofrontal cortices, and periaqueductal gray matter, in which regions constitute the endogenous pain inhibitory pathway (García-Larrea et al., 1999; Garcia-Larrea and Peyron, 2007). A meta-analysis reports that non-invasive motor cortex stimulation could effectively modulate pain thresholds and conditioned pain modulation (CPM) efficiency in the healthy and chronic pain populations (Giannoni-Luza et al., 2020). The CPM paradigm is commonly employed to examine the function and integrity of the endogenous pain inhibitory pathway (Bannister and Dickenson, 2017). Indeed, two recent studies confirmed that the single-session HD-tDCS targeted on the M1 could improve CPM efficiency among healthy populations (Wan et al., 2021; Jiang et al., 2022). Therefore, the observed analgesic effects on pain threshold could have arisen from the top-down modulation of endogenous pain inhibitory pathway *via* HD-tDCS stimulating M1. Nevertheless, we did not measure neurophysiological data that could allow us to assess the mechanisms underlying the analgesic effects. Future neuroimaging studies are recommended to further explore whether and how HD-tDCS over M1 modulates the endogenous pain inhibitory pathway and subjective pain perception.

High-definition tDCS over M1 did not modulate cold pain tolerance and pain intensity and unpleasantness ratings. Human pain is a subjective and multidimensional experience involving sensory-discriminative, affective-motivational, and cognitive-evaluation aspects (Wiech et al., 2008; Tracey, 2011). Although pain threshold, pain tolerance, pain intensity, and unpleasantness ratings are indices of subjective pain perception, they could be reflecting different aspects of pain processing. Pain threshold seems to be determined predominantly by physiological factors, thereby, largely reflecting the sensory-discriminative aspect of pain that is mediated by the primary and secondary somatosensory cortices (Schnitzler and Ploner, 2000; Price et al., 2001; Vierck et al., 2013). Pain tolerance and unpleasantness rating are mainly reflecting the affective-motivational aspect of pain, which is processed in the medial nociceptive system including the anterior cingulate cortex and insula (Peyron et al., 2000; Schnitzler and Ploner, 2000; Bushnell et al., 2013). In contrast, pain intensity rating would be more complex, therefore, encompassing multiple aspects of pain that are encoded in brain regions associated with somatosensory, emotional, attention, and motor processing (Rainville et al., 1992; Coghill et al., 1999). Here, anodal HD-tDCS on M1 increased cold pain threshold but did not significantly affect cold pain tolerance, intensity, and unpleasantness rating. It suggests that HD-tDCS can effectively modulate the sensory-discriminative processes of pain perception, instead of the affective-cognitive aspect of pain perception.

Correlation analysis showed that analgesic effects of anodal HD-tDCS on pain intensity ratings were strongly associated with the participant’s negative attentional bias. It is manifested that those healthy participants with a lower level of attentional bias to negative information tend to have greater effects of HD-tDCS on relieving pain intensity. With the knowledge that patients with chronic pain have more negative attitudes and beliefs, such as pain catastrophizing and fear of pain, as well as negative bias toward pain (Vlaeyen and Linton, 2000; Keogh et al., 2004; Bushnell et al., 2013; Meints and Edwards, 2018), the affective turning of the attentional system could direct attention to negatively-valanced information more frequently and consequently, leading to poor responses to pharmacological interventions of pain (Mankovsky et al., 2012; Burns et al., 2017; Sharifzadeh et al., 2017). For instance, chronic pain patients with less pain catastrophizing or fear of pain tend to exhibit better treatment outcomes (Werneke et al., 2009; Sparkes et al., 2015). Consistent with this understanding, hypervigilance to negative information may counteract the pain-relieving effects induced by HD-tDCS. It suggests that the application of tDCS on relieving clinical pain should well consider the patients’ cognitions, such as attentional bias. Individuals with less attentional bias to negative information could be the ones that can benefit more from the tDCS intervention. This can help guide more precise tDCS intervention in clinical pain management.

## Conclusion

In conclusion, relative to sham stimulation, single-session anodal HD-tDCS over M1 increased cold pain threshold in the healthy population. It indicates anodal HD-tDCS targeted on the M1 can effectively modulate the sensory-discriminative aspect of pain perception. In addition, the effectiveness of anodal HD-tDCS in attenuating pain intensity ratings to suprathreshold pain could be influenced by the level of attentional bias to negative information. Our findings support the potential application of HD-tDCS interventions in pain relief among the clinical pain patients and highlight that individual attentional bias to negative information should be well taken into consideration. Given that multiple-session tDCS may be more effective than single-session stimulation (Monte-Silva et al., 2013; Lefaucheur et al., 2017), future studies are recommended to test the effectiveness on relieving clinical pain through applying HD-tDCS with repeated sessions.

## Data Availability Statement

The original contributions presented in the study are included in the article/supplementary material, further inquiries can be directed to the corresponding author/s.

## Ethics Statement

The studies involving human participants were reviewed and approved by the Medical Ethics Committee, Health Science Center, Shenzhen University. The patients/participants provided their written informed consent to participate in this study.

## Author Contributions

XYL and RJ: conception and design of the study and drafting the manuscript. XYL and JY: data acquisition. XYL and XXL: data analysis. XYL, SC, YH, JL, and RJ: writing—reviewing and editing. All authors read and approved the final manuscript.

## Conflict of Interest

The authors declare that the research was conducted in the absence of any commercial or financial relationships that could be construed as a potential conflict of interest.

## Publisher’s Note

All claims expressed in this article are solely those of the authors and do not necessarily represent those of their affiliated organizations, or those of the publisher, the editors and the reviewers. Any product that may be evaluated in this article, or claim that may be made by its manufacturer, is not guaranteed or endorsed by the publisher.

## References

[B1] Antal A. Terney D. Kühnl S. Paulus W. (2010). Anodal transcranial direct current stimulation of the motor cortex ameliorates chronic pain and reduces short intracortical inhibition. *J. Pain Symptom Manage.* 39 890–903. 10.1016/j.jpainsymman.2009.09.023 20471549

[B2] Bachmann C. G. Muschinsky S. Nitsche M. A. Rolke R. Magerl W. Treede R. D. (2010). Transcranial direct current stimulation of the motor cortex induces distinct changes in thermal and mechanical sensory percepts. *Clin. Neurophysiol.* 121 2083–2089. 10.1016/j.clinph.2010.05.005 20570558

[B3] Bannister K. Dickenson A. H. (2017). The plasticity of descending controls in pain: translational probing. *J. Physiol.* 595 4159–4166. 10.1113/jp274165 28387936 PMC5491855

[B4] Bikson M. Datta A. Rahman A. Scaturro J. (2010). Electrode montages for tDCS and weak transcranial electrical stimulation: role of “return” electrode’s position and size. *Clin. Neurophysiol.* 121 1976–1978. 10.1016/j.clinph.2010.05.020 21035740 PMC2983105

[B5] Borckardt J. J. Bikson M. Frohman H. Reeves S. T. Datta A. Bansal V. (2012). A pilot study of the tolerability and effects of high-definition transcranial direct current stimulation (HD-tDCS) on pain perception. *J. Pain* 13 112–120. 10.1016/j.jpain.2011.07.001 22104190

[B6] Brasil-Neto J. P. Iannone A. Caixeta F. V. Cavendish B. A. de Mello Cruz A. P. Buratto L. G. (2020). Acute offline transcranial direct current stimulation does not change pain or anxiety produced by the cold pressor test. *Neurosci. Lett.* 736:135300. 10.1016/j.neulet.2020.135300 32781010

[B7] Brunoni A. R. Amadera J. Berbel B. Volz M. S. Rizzerio B. G. Fregni F. (2011). A systematic review on reporting and assessment of adverse effects associated with transcranial direct current stimulation. *Int. J. Neuropsychopharmacol.* 14 1133–1145. 10.1017/s1461145710001690 21320389

[B8] Burns J. W. Bruehl S. France C. R. Schuster E. Orlowska D. Buvanendran A. (2017). Psychosocial factors predict opioid analgesia through endogenous opioid function. *Pain* 158 391–399. 10.1097/j.pain.0000000000000768 27898491 PMC7176103

[B9] Bushnell M. C. Ceko M. Low L. A. (2013). Cognitive and emotional control of pain and its disruption in chronic pain. *Nat. Rev. Neurosci.* 14 502–511. 10.1038/nrn3516 23719569 PMC4465351

[B10] Castillo-Saavedra L. Gebodh N. Bikson M. Diaz-Cruz C. Brandao R. Coutinho L. (2016). Clinically effective treatment of fibromyalgia pain with high-definition transcranial direct current stimulation: phase II open-label dose optimization. *J. Pain* 17 14–26. 10.1016/j.jpain.2015.09.009 26456677 PMC5777157

[B11] Coghill R. C. Sang C. N. Maisog J. M. Iadarola M. J. (1999). Pain intensity processing within the human brain: a bilateral, distributed mechanism. *J. Neurophysiol.* 82 1934–1943. 10.1152/jn.1999.82.4.1934 10515983

[B12] Creutzfeldt O. D. Fromm G. H. Kapp H. (1962). Influence of transcortical dc currents on cortical neuronal activity. *Exp. Neurol.* 5 436–452. 10.1016/0014-4886(62)90056-0 13882165

[B13] Datta A. Bansal V. Diaz J. Patel J. Reato D. Bikson M. (2009). Gyri-precise head model of transcranial direct current stimulation: improved spatial focality using a ring electrode versus conventional rectangular pad. *Brain Stimul.* 2 201–207, 207.e201. 10.1016/j.brs.2009.03.005 20648973 PMC2790295

[B14] Edwards D. Cortes M. Datta A. Minhas P. Wassermann E. M. Bikson M. (2013). Physiological and modeling evidence for focal transcranial electrical brain stimulation in humans: a basis for high-definition tDCS. *Neuroimage* 74 266–275. 10.1016/j.neuroimage.2013.01.042 23370061 PMC4359173

[B15] Ehrlich P. F. Vedulla G. Cottrell N. Seidman P. A. (2003). Monitoring intraoperative effectiveness of caudal analgesia through skin temperature variation. *J. Pediatr. Surg.* 38 386–389. 10.1053/jpsu.2003.50113 12632354

[B16] Fregni F. Freedman S. Pascual-Leone A. (2007). Recent advances in the treatment of chronic pain with non-invasive brain stimulation techniques. *Lancet Neurol.* 6 188–191. 10.1016/s1474-4422(07)70032-7 17239806

[B17] Garcia-Larrea L. Peyron R. (2007). Motor cortex stimulation for neuropathic pain: from phenomenology to mechanisms. *Neuroimage* 37 Suppl 1 S71–S79. 10.1016/j.neuroimage.2007.05.062 17644413

[B18] García-Larrea L. Peyron R. Mertens P. Gregoire M. C. Lavenne F. Le Bars D. (1999). Electrical stimulation of motor cortex for pain control: a combined PET-scan and electrophysiological study. *Pain* 83 259–273. 10.1016/s0304-3959(99)00114-1 10534598

[B19] Giannoni-Luza S. Pacheco-Barrios K. Cardenas-Rojas A. Mejia-Pando P. F. Luna-Cuadros M. A. Barouh J. L. (2020). Noninvasive motor cortex stimulation effects on quantitative sensory testing in healthy and chronic pain subjects: a systematic review and meta-analysis. *Pain* 161 1955–1975. 10.1097/j.pain.0000000000001893 32453135 PMC7679288

[B20] Grundmann L. Rolke R. Nitsche M. A. Pavlakovic G. Happe S. Treede R. D. (2011). Effects of transcranial direct current stimulation of the primary sensory cortex on somatosensory perception. *Brain Stimul.* 4 253–260. 10.1016/j.brs.2010.12.002 22032740

[B21] Jiang X. Wang Y. Wan R. Feng B. Zhang Z. Lin Y. (2022). The effect of high-definition transcranial direct current stimulation on pain processing in a healthy population: a single-blinded crossover controlled study. *Neurosci. Lett.* 767:136304. 10.1016/j.neulet.2021.136304 34695451

[B22] Jürgens T. P. Schulte A. Klein T. May A. (2012). Transcranial direct current stimulation does neither modulate results of a quantitative sensory testing protocol nor ratings of suprathreshold heat stimuli in healthy volunteers. *Eur. J. Pain* 16 1251–1263. 10.1002/j.1532-2149.2012.00135.x 22416036

[B23] Keogh E. Hamid R. Hamid S. Ellery D. (2004). Investigating the effect of anxiety sensitivity, gender and negative interpretative bias on the perception of chest pain. *Pain* 111 209–217. 10.1016/j.pain.2004.06.017 15327825

[B24] Kold S. Graven-Nielsen T. (2021). Effect of anodal high-definition transcranial direct current stimulation on the pain sensitivity in a healthy population: a double-blind, sham-controlled study. *Pain* 162 1659–1668. 10.1097/j.pain.0000000000002187 33449508

[B25] Kuo H. I. Bikson M. Datta A. Minhas P. Paulus W. Kuo M. F. (2013). Comparing cortical plasticity induced by conventional and high-definition 4 × 1 ring tDCS: a neurophysiological study. *Brain Stimul.* 6 644–648. 10.1016/j.brs.2012.09.010 23149292

[B26] Lang N. Siebner H. R. Ward N. S. Lee L. Nitsche M. A. Paulus W. (2005). How does transcranial DC stimulation of the primary motor cortex alter regional neuronal activity in the human brain? *Eur. J. Neurosci.* 22 495–504. 10.1111/j.1460-9568.2005.04233.x 16045502 PMC3717512

[B27] Lefaucheur J. P. Antal A. Ayache S. S. Benninger D. H. Brunelin J. Cogiamanian F. (2017). Evidence-based guidelines on the therapeutic use of transcranial direct current stimulation (tDCS). *Clin. Neurophysiol.* 128 56–92. 10.1016/j.clinph.2016.10.087 27866120

[B28] Lefaucheur J.-P. Antal A. Ahdab R. de Andrade D. C. Fregni F. Khedr E. M. (2008). The use of repetitive transcranial magnetic stimulation (rTMS) and transcranial direct current stimulation (tDCS) to relieve pain. *Brain Stimul.* 1 337–344. 10.1016/j.brs.2008.07.003 20633392

[B29] Luedtke K. Rushton A. Wright C. Geiss B. Juergens T. P. May A. (2012b). Transcranial direct current stimulation for the reduction of clinical and experimentally induced pain: a systematic review and meta-analysis. *Clin. J. Pain* 28 452–461. 10.1097/AJP.0b013e31823853e3 22569218

[B30] Luedtke K. May A. Jürgens T. P. (2012a). No effect of a single session of transcranial direct current stimulation on experimentally induced pain in patients with chronic low back pain–an exploratory study. *PLoS One* 7:e48857. 10.1371/journal.pone.0048857 23189136 PMC3506580

[B31] Luedtke K. Rushton A. Wright C. Jürgens T. Polzer A. Mueller G. (2015). Effectiveness of transcranial direct current stimulation preceding cognitive behavioural management for chronic low back pain: sham controlled double blinded randomised controlled trial. *BMJ* 350 h1640. 10.1136/bmj.h1640 25883244 PMC4399394

[B32] Mankovsky T. Lynch M. Clark A. Sawynok J. Sullivan M. J. (2012). Pain catastrophizing predicts poor response to topical analgesics in patients with neuropathic pain. *Pain Res. Manag.* 17 10–14. 10.1155/2012/970423 22518362 PMC3299037

[B33] McCracken L. M. (1997). Attention to pain in persons with chronic pain: a behavioral approach. *Behav. Ther.* 28 271–284. 10.1016/S0005-7894(97)80047-0

[B34] McNeil D. W. Rainwater A. J. (1998). Development of the fear of pain questionnaire-III. *J. Behav. Med.* 21 389–410. 10.1023/a:1018782831217 9789168

[B35] Meints S. M. Edwards R. R. (2018). Evaluating psychosocial contributions to chronic pain outcomes. *Prog. Neuropsychopharmacol. Biol. Psychiatry* 87(Pt B) 168–182. 10.1016/j.pnpbp.2018.01.017 29408484 PMC6067990

[B36] Minhas P. Bansal V. Patel J. Ho J. S. Diaz J. Datta A. (2010). Electrodes for high-definition transcutaneous DC stimulation for applications in drug delivery and electrotherapy, including tDCS. *J. Neurosci. Methods* 190 188–197. 10.1016/j.jneumeth.2010.05.007 20488204 PMC2920288

[B37] Monte-Silva K. Kuo M. F. Hessenthaler S. Fresnoza S. Liebetanz D. Paulus W. (2013). Induction of late LTP-like plasticity in the human motor cortex by repeated non-invasive brain stimulation. *Brain Stimul.* 6 424–432. 10.1016/j.brs.2012.04.011 22695026

[B38] Mylius V. Borckardt J. J. Lefaucheur J. P. (2012). Noninvasive cortical modulation of experimental pain. *Pain* 153 1350–1363. 10.1016/j.pain.2012.04.009 22633979

[B39] Nitsche M. A. Paulus W. (2000). Excitability changes induced in the human motor cortex by weak transcranial direct current stimulation. *J. Physiol.* 527(Pt 3) 633–639. 10.1111/j.1469-7793.2000.t01-1-00633.x 10990547 PMC2270099

[B40] Nitsche M. A. Cohen L. G. Wassermann E. M. Priori A. Lang N. Antal A. (2008). Transcranial direct current stimulation: state of the art 2008. *Brain Stimul.* 1 206–223. 10.1016/j.brs.2008.06.004 20633386

[B41] Nitsche M. A. Doemkes S. Karaköse T. Antal A. Liebetanz D. Lang N. (2007). Shaping the effects of transcranial direct current stimulation of the human motor cortex. *J. Neurophysiol.* 97 3109–3117. 10.1152/jn.01312.2006 17251360

[B42] Noguchi K. Gohm C. L. Dalsky D. J. (2006). Cognitive tendencies of focusing on positive and negative information. *J. Res. Pers.* 40 891–910. 10.1016/j.jrp.2005.09.008

[B43] Peyron R. Laurent B. Garcia-Larrea L. (2000). Functional imaging of brain responses to pain. A review and meta-analysis (2000). *Neurophysiol. Clin.* 30 263–288. 10.1016/s0987-7053(00)00227-6 11126640

[B44] Price D. D. Riley J. L. Wade J. B. (2001). “Psychophysical approaches to measurement of the dimensions and stages of pain,” in *Handbook Of Pain Assessment*, 2nd Edn, eds Turk D. C. Melzack R. (New York, NY: The Guilford Press), 53–75. 10.1097/PR9.0000000000000821

[B45] Purpura D. P. McMurtry J. G. (1965). Intracellular activities and evoked potential changes during polarization of motor cortex. *J. Neurophysiol.* 28 166–185. 10.1152/jn.1965.28.1.166 14244793

[B46] Rainville P. Feine J. S. Bushnell M. C. Duncan G. H. (1992). A psychophysical comparison of sensory and affective responses to four modalities of experimental pain. *Somatosens. Mot. Res.* 9 265–277. 10.3109/08990229209144776 1492527

[B47] Ruscheweyh R. Marziniak M. Stumpenhorst F. Reinholz J. Knecht S. (2009). Pain sensitivity can be assessed by self-rating: development and validation of the Pain Sensitivity Questionnaire. *Pain* 146 65–74. 10.1016/j.pain.2009.06.020 19665301

[B48] Schnitzler A. Ploner M. (2000). Neurophysiology and functional neuroanatomy of pain perception. *J. Clin. Neurophysiol.* 17 592–603. 10.1097/00004691-200011000-00005 11151977

[B49] Sharifzadeh Y. Kao M. C. Sturgeon J. A. Rico T. J. Mackey S. Darnall B. D. (2017). Pain catastrophizing moderates relationships between pain intensity and opioid prescription: nonlinear sex differences revealed using a learning health system. *Anesthesiology* 127 136–146. 10.1097/aln.0000000000001656 28614083 PMC5478434

[B50] Sparkes E. Duarte R. V. Mann S. Lawrence T. R. Raphael J. H. (2015). Analysis of psychological characteristics impacting spinal cord stimulation treatment outcomes: a prospective assessment. *Pain Phys.* 18 E369–E377. 26000684

[B51] Sullivan M. J. Bishop S. R. Pivik J. (1995). The pain catastrophizing scale: development and validation. *Psychol. Assess.* 7 524–532. 10.1037/1040-3590.7.4.524

[B52] Tracey I. (2011). Can neuroimaging studies identify pain endophenotypes in humans? *Nat. Rev. Neurol.* 7 173–181. 10.1038/nrneurol.2011.4 21304481

[B53] Vaseghi B. Zoghi M. Jaberzadeh S. (2014). Does anodal transcranial direct current stimulation modulate sensory perception and pain? A meta-analysis study. *Clin. Neurophysiol.* 125 1847–1858. 10.1016/j.clinph.2014.01.020 24555922

[B54] Vaseghi B. Zoghi M. Jaberzadeh S. (2015). A meta-analysis of site-specific effects of cathodal transcranial direct current stimulation on sensory perception and pain. *PLoS One* 10:e0123873. 10.1371/journal.pone.0123873 25978673 PMC4433259

[B55] Vierck C. J. Whitsel B. L. Favorov O. V. Brown A. W. Tommerdahl M. (2013). Role of primary somatosensory cortex in the coding of pain. *Pain* 154 334–344. 10.1016/j.pain.2012.10.021 23245864 PMC4501501

[B56] Villamar M. F. Volz M. S. Bikson M. Datta A. Dasilva A. F. Fregni F. (2013). Technique and considerations in the use of 4x1 ring high-definition transcranial direct current stimulation (HD-tDCS). *J. Vis. Exp.* 77:e50309. 10.3791/50309 23893039 PMC3735368

[B57] Vlaeyen J. W. S. Linton S. J. (2000). Fear-avoidance and its consequences in chronic musculoskeletal pain: a state of the art. *Pain* 85 317–332. 10.1016/s0304-3959(99)00242-0 10781906

[B58] Wan R. Wang Y. Feng B. Jiang X. Xu Y. Zhang Z. (2021). Effect of high-definition transcranial direct current stimulation on conditioned pain modulation in healthy adults: a crossover randomized controlled trial. *Neuroscience* 479 60–69. 10.1016/j.neuroscience.2021.10.019 34710538

[B59] Werneke M. W. Hart D. L. George S. Z. Stratford P. W. Matheson J. W. Reyes A. (2009). Clinical outcomes for patients classified by fear-avoidance beliefs and centralization phenomenon. *Arch. Phys. Med. Rehabil.* 90 768–777. 10.1016/j.apmr.2008.11.008 19406296

[B60] Wiech K. Ploner M. Tracey I. (2008). Neurocognitive aspects of pain perception. *Trends Cogn. Sci.* 12 306–313. 10.1016/j.tics.2008.05.005 18606561

[B61] Zandieh A. Parhizgar S. E. Fakhri M. Taghvaei M. Miri S. Shahbabaie A. (2013). Modulation of cold pain perception by transcranial direct current stimulation in healthy individuals. *Neuromodulation* 16 345–348; discussion348. 10.1111/ner.12009 23240605

